# Chronic unpredictable stress induces autophagic death of adult hippocampal neural stem cells

**DOI:** 10.1186/s13041-024-01105-6

**Published:** 2024-06-03

**Authors:** Seongwon Choe, Hyeonjeong Jeong, Jieun Choi, Seong-Woon Yu

**Affiliations:** Department of Brain Sciences, Daegu Gyeongbuk Institute of Science and Technology (DGIST), 333 Techno Jungang Daero, Hyeonpung-Myeon, Dalseong-Gun, Daegu, 42988 Republic of Korea

## Abstract

Chronic psychological stress is a critical factor for neurological complications like anxiety disorders, dementia, and depression. Our previous results show that chronic restraint stress causes cognitive deficits and mood dysregulation by inducing autophagic death of adult hippocampal neural stem cells (NSCs). However, it is unknown whether other models of psychological stress also induce autophagic death of adult hippocampal NSCs. Here, we show that chronic unpredictable stress (CUS) for 10 days impaired memory function and increased anxiety in mice. Immunohistochemical staining with SOX2 and KI67 revealed a significant reduction in the number of NSCs in the hippocampus following exposure to CUS. However, these deficits were prevented by NSC-specific, inducible conditional deletion of *Atg7*. These findings suggest that autophagic death of adult hippocampal NSCs is a critical pathogenic mechanism underlying stress-induced brain disorders.

**Supplementary Information:**

The online version contains supplementary material available at 10.1186/s13041-024-01105-6.

## Main text

Chronic psychological stress has emerged as a key determinant in the development and progression of various neurological complications, including anxiety disorders, dementia, and depression [1–3]. Recent studies have shown that prolonged exposure to psychological stress can disrupt normal neurobiological processes, leading to structural and functional changes in key brain regions involved in emotional regulation, cognition, and memory [4, 5].

Autophagy is a highly regulated intracellular mechanism responsible for maintenance of cellular homeostasis by removing damaged organelles, protein aggregates, and other cellular debris [6]. In contrast to this well-established homeostatic and cytoprotective roles of autophagy, autophagy can also function as one of the modes of programmed cell death through the degradation of key cellular components [7]. Autophagic death has been implicated in various physiological and pathological contexts, including the response to cellular stressors such as nutrient deprivation, hypoxia, or exposure to toxins [8].

We previously reported that chronic restraint stress (CRS) leads to cognitive deficits and mood dysregulation by inducing autophagic death of adult hippocampal neural stem cells (NSCs), highlighting the potential impact of stress on the regenerative capacity of the brain [9].

In the past, programmed cell death has not been considered as the mechanisms underlying stress-induced hippocampal damage and cognitive impairments, due to the lack of the evidence of apoptosis [10, 11]. Consistent with those previous reports, CRS did not induce caspase 3 activation or chromosomal DNA fragmentation, well-known apoptotic markers [9]. Instead, CRS increased autophagy flux in adult hippocampal NSCs and NSC-specific, inducible deletion of *Atg7*, one of the key autophagy genes, prevented CRS-induced death of adult hippocampal NSCs and preserved cognitive functions [9]. These findings suggest that CRS suppresses adult hippocampal neurogenesis by triggering autophagic death of adult hippocampal NSCs and provide a new perspective on neurobiological mechanisms and potential therapeutic strategies for stress-related neurological disorders. However, it remains unknown whether other models of psychological stress elicit similar effects on adult hippocampal neurogenesis through autophagic death of NSCs.

**Fig. 1:**
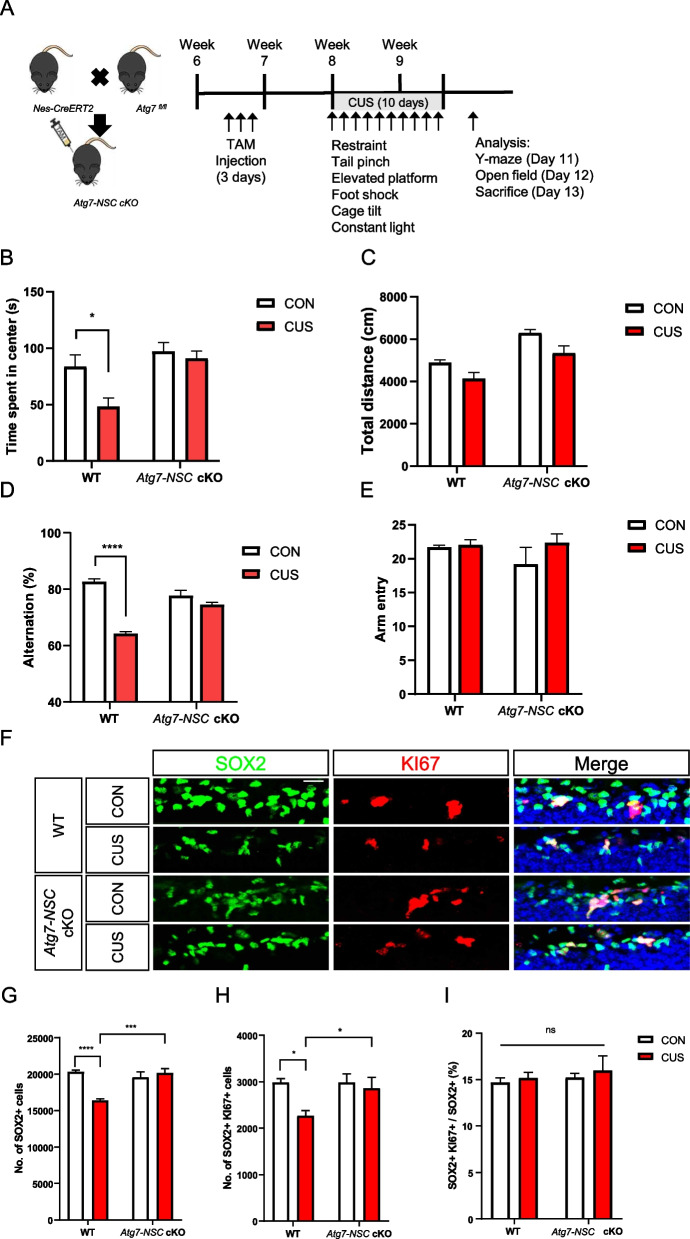
NSC-specific deletion of *Atg7* prevents CUS-induced anxiety-like behavior, memory deficit, and the reduction in the number of adult hippocampal NSCs. **A** Experimental schedule of CUS given for 10 days. **B**, **C** Open field test. Time spent in the center (**B**) and total distance (**C**) in the open field test. **D**, **E** Y-maze test. Arm alternation (**D**) and total arm entry (**E**) in the Y-maze test. **F** Representative images of SOX2 and KI67 staining in the subgranular zone of the hippocampus. Scale bar: 20 μm. **G**-**I** Quantification of SOX2^+^ and KI67^+^ cells. Number of SOX2 + cells (**G**), SOX2 + KI67 + cells (**H**) and the ratio of SOX2 + KI67 + to SOX2 + cells (**I**) (*n* = 6 or 7 per group). *P < 0.05, *** *P* < 0.001, **** *P* < 0.0001. ns, not significant

To address this question, mice were subject to chronic unpredictable stress (CUS) for 10 days (Table 1) and we examined the cellular and behavioral changes of mice caused by CUS and the role of *Atg7* in CUS-induced loss of adult hippocampal NSCs. First, to generate mice with an inducible NSC-targeting *Atg7* conditional knockout (*Atg7-NSC* cKO) mice, *Atg7*^fl/fl^ mice were crossed with *nestin-CreERT2* (*Nes-Cre*). In our previous study, we induced heterozygous ablation of *Atg7* to avoid compounding effects of prolonged suppression of autophagy [9]. To induce *Atg7* conditional knockout at the adult stage, 7-week-old *Atg7*^fl/+^ (WT) or *nestin-CreERT2: Atg7*^fl/+^ (*Atg7-NSC* cKO) mice were intraperitonially (i.p.) injected with tamoxifen (TAM) for 3 days and CUS was performed 1 week later. For 10 days, mice were exposed to one of six types of stresses daily, as detailed in Table 1. To investigate whether NSC-specific deletion of *Atg7* keeps brain function intact, adult neurogenesis-dependent hippocampal functions were tested. After CUS, WT mice spent shorter time in the center of the open field in the open field test (Fig. 1B) and open arms in the elevated maze test (Figure S1A) and showed less spontaneous arm alternation in the Y-maze test (Fig. 1D) and decreased discrimination index in the novel object location test (Figure S1C), suggesting anxiety-like behaviors and spatial memory impairment by CUS. However, stressed *Atg7-NSC* cKO mice were resistant to CUS and did not show these neurobehavioral alterations (Fig. 1B, D, Figure S1A, C). There was no difference between WT and *Atg7-NSC* cKO groups in the total traveled distance in open field test (Fig. 1C) and total number of arm entries in Y-mase (Fig. 1E) and elevated plus maze tests (Figure S1B). To measure the death of NSCs, we counted the number of total (SOX2-positive) and proliferating NSCs (KI67/SOX2 double-positive) after CUS (Fig. 1F). CUS significantly decreased the numbers of total (Fig. 1G) and proliferating (Fig. 1H) NSCs in WT mice. Of note, the ratio of the proliferating NSCs was not affected by CUS, confirming that the decrease in the number of NSCs was due to death of NSCs rather than inhibition of proliferation (Fig. 1I). In contrast, *Atg7* ablation blocked death of NSCs and prevented CUS-induced decrease in the number of NSCs (Fig. 1F-I). To demonstrate an increase in autophagy flux in vivo, we performed stereotaxic injection of *Nestin* promoter-driven mRFP-EGFP-MAP1LC3B lentivirus in mouse hippocampus, as we previously performed [9]. This tandem MAP1LC3B construct demonstrated a higher ratio of autolysosomes and an increase in the total number of MAP1LC3B puncta in WT hippocampal NSCs following CUS (Figure S1D-E), suggesting an increase in autophagy flux. However, these increases were not observed in *Atg7-NSC* cKO mice (Figure S1D-E). In contrast to the induction of autophagy flux, we were unable to detect activated caspase-3 (cleaved caspase-3, c.CASP3, Figure S1F) and TUNEL signals (Figure S1G) following CUS. As positive control, staurosporine (STS) was stereotaxically injected for the induction of CASP3 activation and DNA damage (Figure S1F-G). These results indicate the reduction of hippocampal NSCs by autophagy but not apoptosis, following CUS.

**Table 1:** A schedule for chronic unpredictable stress for 10 days

Day	Type of stressor	Time
1	2 h restraint	10 a.m
2	15 min tail pinch^a^	3 p.m
3	30 min elevated platform	10 a.m
4	16 h constant light	Starting at 7:00 p.m
5	10 min inescapable foot shocks^b^	10 a.m
6	30 min elevated platform	3 p.m
7	15 min tail pinch^a^	10 a.m
8	2 h restraint	3 p.m
9	24 h wet bedding with 45° cage tilt	Starting at 7:00 a.m
10	10 min inescapable foot shocks^b^	3 p.m

^a^Tail pinch: pinching the tail to the edge of the homecage using clothing clip, just leaving the foreleg touching the ground

^b^Electric shock: 10 min, 0.3 mA, 2 s duration with the interval of 16 s

Chronic stress can increase the risk of developing depression and relapse by inhibiting the production of new neurons [12]. Current treatments for depression and stress-related neurological disorders requires long-term medication and are not effective for all patients, and some patients may have drug resistance or experience side effects [13]. Therefore, it is imperative to find new treatment targets to improve the treatment effectiveness of depression and stress-related neurological disorders.

Our results demonstrate that *Atg7-NSC* cKO mice are functionally intact by prevention of autophagic death of adult hippocampal NSCs. Understanding the role of autophagic death in the context of chronic stress can provide insights into the mechanisms underlying stress-induced brain disorders and potentially guide the development of new therapeutic strategies to mitigate their impact.

## Methods

### Mice

*Nestin-CreERT2* transgenic mice were purchased from The Jackson Laboratory and *Atg7*^*fl/fl*^ mice were provided by Dr. Myung-Shik Lee (Yonsei University, Korea) with the permission of Dr. Massaki Komatsu (Tokyo Metropolitan Institute of Medical Science, Japan). *Nestin-CreERT2* mice were crossed with *Atg7*^*fl/fl*^ mice to generate TAM-inducible, NSC-specific, heterozygous conditional knockout mice of *Atg7*.

### TAM injection

TAM (Sigma, T5648) was dissolved in corn oil (Sigma, C8267) with sonication at a concentration of 30 mg/ml until particles were no longer detectable, and was stored at –20 °C. Before injection, TAM was incubated at 80 °C and 7-week-old *Atg7*^fl/+^ or *Nestin-CreERT2:Atg7*^fl/+^ mice were i.p. injected with 180 mg/kg of TAM three times with a 24-h interval.

### Open field test

The apparatus consisted of a square-shaped arena (40 × 40 cm^2^). Mice were placed facing the center of one of the walls and allowed to explore the apparatus for 20 min. The time spent exploring the central region (20 × 20 cm^2^ area) and total distance traveled were measured. Data were collected using EthoVision Observer.

### Y-maze test

The Y-maze was constructed of white plastic with three arms that extended from a central platform at a 120° angle. Each mouse was placed in the center of the Y-maze and was allowed to explore freely through the maze for 6 min. The sequence and total number of arms entered were recorded using EthoVision Observer. Arm entry was considered to complete when the whole body of the mouse was completely placed within the arm.

### Immunohistochemistry

After behavior test, mice were anaesthetized with Zoletil (Virbac, 6FX7; 50 mg/kg) and Rompun (Bayer; 10 mg/kg) and perfused with full name (PBS), followed by 4% full name (PFA). Brains were dissected and post-fixed in 4% PFA for 12 h at 4 °C. Brains were then cryoprotected in 30% sucrose (wt/vol) in PBS at 4 °C until they sank to the bottom of the tube and subsequently frozen in optimum cutting temperature (OCT) compound and stored at -80 °C until cryostat sectioning. Frozen block was cut into 40-µm thick coronal sections. Samples were free-floated in PBS and blocked with PBS containing 1% bovine serum albumin and 0.3% Triton X-100 for 1 h at room temperature. After blocking, samples were then incubated with the appropriate primary antibodies (Supplementary Table S1) for 24 h at 4 °C and washed three times for 10 min. Appropriated secondary antibodies were used and incubated in Hoechst33342 dye (1: 10,000) in PBS for 10 min. Sections were then mounted onto glass slide and cover slipped with Fluorescence Mounting Medium (Dako) for microscopic analysis.

### Stereology

The dentate gyrus from -1.2 mm to -2.8 mm of Bregma and the immunoreactive cells of both hemispheres were counted by dividing the 40-um coronal sections into five stages. The cells were quantified using the optical fractionator method of Stereo Investigator software (MBF Bioscience). For the representative images, medial section of dentate gyrus (bregma − 2.0 mm) was imaged with LSM 780 confocal microscope (Carl Zeiss).

### Statistics

Data were expressed as the mean ± standard error of the mean (SEM) of at least three independent experiments. All analyses were performed with the experimenter blinded to the groups of mice. Statistical significance was determined using two-way ANOVA test followed by Bonferroni’s multiple comparison test. Sample sizes were selected based on preliminary results to ensure proper power. Differences were considered statistically significant at *P* < 0.05.

### Supplementary Information

Supplementary Material 1.

## Data Availability

All data generated during this study are included in this article.
