# Supplementary material for: Chronic unpredictable stress induces autophagic death of adult hippocampal neural stem cells
Source: Mol Brain. 2024 Jun 3;17:31. doi: 10.1186/s13041-024-01105-6 (PMC11145853; doi:10.1186/s13041-024-01105-6)
Supplement: Supplementary file 1 — Supplementary Material 1. [file 13041_2024_1105_MOESM1_ESM.docx]

**Additional file 1**

**Chronic unpredictable stress induces autophagic death of adult hippocampal neural stem cells**

Seongwon Choe, Hyeonjeong Jeong, Jieun Choi and Seong-Woon Yu^*^

Department of Brain Sciences, Daegu Gyeongbuk Institute of Science and Technology (DGIST), Daegu, 42988, Republic of Korea;

* Correspondence Address:

Seong-Woon Yu, Ph.D.

Department of Brain Sciences

Daegu Gyeongbuk Institute of Science & Technology

333 Techno Jungang Daero, Hyeonpung-Myeon, Dalseong-Gun, Daegu 42988, Republic of Korea

Phone: 82-53-785-6113

Fax: 82-53-785-6109

Email: yusw@dgist.ac.kr

This file includes:

Methods

Additional Figure S1

Supplementary Table S1

**Methods**

**Elevated plus maze test**

The maze apparatus consists of a “+”-shaped maze elevated above the floor with two closed arms and two open arms. The mice were placed in the center of the maze facing one of the open arms, and allowed to explore the maze freely for 5 min. And then, the time spent in the open arms was measured. Data were collected using EthoVision Observer.

**Nobel object location test**

Testing was performed in an open field arena (40 × 40 cm^2^), to which the mice were first habituated for 10 min (Day 1). At day 2, two same objects were placed diagonally in opposite corners and the mice were allowed to explore the arena for 10 min (training phase). The next day (test phase), location of one object was moved to the corner of the same side and the mice were reintroduced to the experimental apparatus for 10 min. The time spent on exploration of each object was measured. Data were collected using EthoVision Observer.

**Lentiviral construct and stereotaxic injection**

mRFP-EGFP-MAP1LC3B (21074 from Addgene) was cloned in pEZX-LMP02 (GeneCopoeia, HPRM13080) containing the human *Nes* promoter and lentivirus was produced as previously reported (1). Mice were anesthetized and stereotaxic surgery was performed to deliver 1 μl of lentivirus in the DG (AP -2.2 m, ML +/-1.5 mm, DV -2.1 mm from the bregma) at 0.1 μl/min over period of 20 min by using a 10 μl Hamilton syringe and 27G needle.

**Immunohistochemical detection of cleaved caspase-3 (c.CASP3) and TUNEL assay**

Samples were treated for antigen retrieval with 10 mM sodium citrate solution (pH 6.0) containing 0.05% Tween 20 for 5 min in 95°C water bath and blocked with PBS containing 1% bovine serum albumin and 0.3% Triton X-100 for 1 h at room temperature. After blocking, samples were then incubated with the primary antibi for c.CASP-3 for 24 h at 4°C and examined for microscopic analysis. TUNEL assay was performed using TUNEL assay kit (Promega, G3250) according to the manufacturer’s instruction.

Additional Figure S1
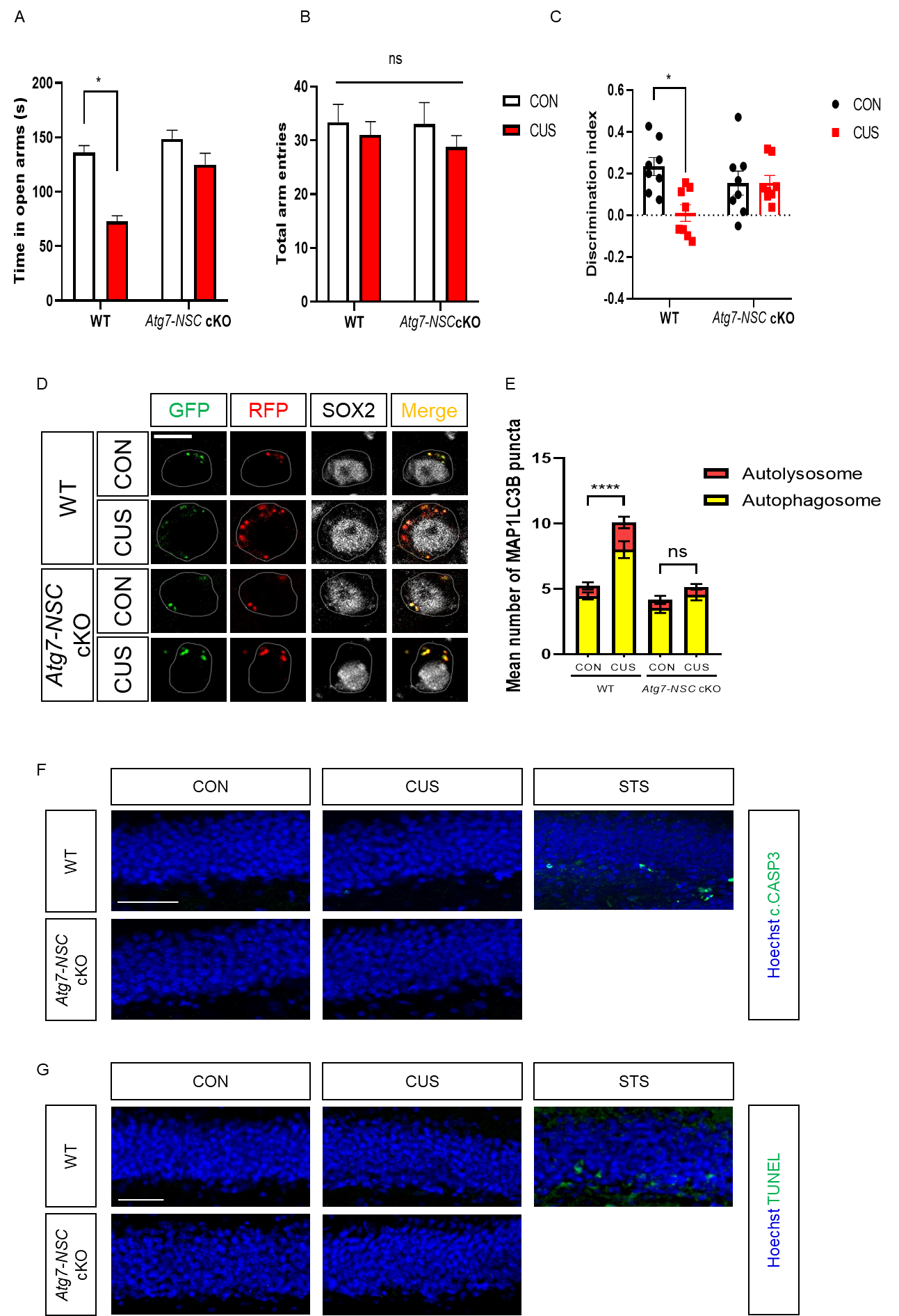


**Figure legend**

Additional Figure S1. CUS induces autophagy, not apoptosis. A-B Elevated plus maze test. Time in open arms (A) and total arm entries (B) in the elevated plus maze test. C Discrimination index in novel object location test (*n* = 6 or 8 per group). D Representative images of *Nes* promoter-driven lentiviral expression of mRFP-EGFP-MRAP1LC3B in the dentate gyrus of the hippocampus*.* Scale bar: 10 μm. E Quantification of autophagosomes and autolysosomes (*n* = 10 or 13 per group). F-G Representative images of cleaved Caspase 3 (c.CASP3) staining (F) and TUNEL assay (G) in the subgranular zone of the hippocampus. Scale bar: 50μm. **P <* 0.05, **** *P* < 0.0001. ns, not significant.

Supplementary Table S1. Antibodies used in this study.

| **Target** | **Species** | **Application** | **Dilution** | **Company and catalog number** |
| --- | --- | --- | --- | --- |
| Ki-67 | Rabbit | IHC | 1:500 | Abcam ab1558 |
| SOX2 | Mouse | IHC | 1:500 | Abcam ab79351 |
| Cleaved caspase 3 | Rabbit | IHC | 1:300 | Cell Signaling Technology 9664 |
| Mouse IgG, Alexa Fluor 488 | Mouse | IHC | 1:500 | Jackson Laboratories 715-545-151 |
| Rabbit IgG, Alexa Fluor 555 | Rabbit | IHC | 1:500 | Themo Fisher Scientific A-31572 |
| Rabbit IgG, Alexa Fluor 488 | Rabbit | IHC | 1:500 | Jackson Laboratories 715-545-152 |

References

1. Jung S, Choe S, Woo H, Jeong H, An H-K, Moon H, et al. Autophagic death of neural stem cells mediates chronic stress-induced decline of adult hippocampal neurogenesis and cognitive deficits. Autophagy. 2020;16(3):512-30.
